# Imaging the operated colon using water-enema multidetector CT, with emphasis on surgical anastomoses

**DOI:** 10.1007/s13244-018-0612-7

**Published:** 2018-04-09

**Authors:** Massimo Tonolini, Sonia Ippolito

**Affiliations:** Department of Radiology, “Luigi Sacco” University Hospital, Via G.B. Grassi 74, 20157 Milan, Italy

**Keywords:** Computed tomography (CT), Colonoscopy, Colorectal surgery, Anastomosis, Stricture, Colorectal carcinoma, Crohn’s disease

## Abstract

**Abstract:**

Water-enema multidetector CT (WE-MDCT) provides a detailed multiplanar visualisation of mural, intra- and extraluminal abnormalities of the large bowel, relying on preliminary bowel cleansing, retrograde luminal distension, pharmacological hypotonisation and intravenous contrast enhancement. In patients with a history of colorectal surgery for either carcinoma or Crohn’s disease (CD), WE-MDCT may also be performed via a colostomy, which allows depicting the anatomy and position of the residual large bowel and evaluates the calibre, length, mural and extraluminal features of luminal strictures. Therefore, WE-MDCT may prove useful as a complementary technique after incomplete or inconclusive colonoscopy to assess features and suspected abnormalities of the surgical anastomosis, particularly when endoscopic or surgical interventions are being planned. This pictorial essay presents the WE-MDCT technique and pitfalls, the expected appearances after different colic surgeries and the imaging features of benign anastomotic disorders (fibrotic stricture, kinking, inflammatory ulcer) and of locally recurrent tumours and CD.

**Teaching points:**

• *Water-enema multidetector CT (WE-MDCT) effectively visualises the operated colon*

• *Complementary to endoscopy, WE-MDCT may helpfully depict abnormalities of surgical anastomoses*

• *WE-MDCT allows assessment of strictures’ features and abnormalities of the upstream bowel*

• *Technical pitfalls, normal postsurgical findings and benign anastomotic disorders are presented*

• *WE-MDCT allows detecting relapsing Crohn’s disease, recurrent and metachronous tumours*

## Introduction

Since the first description by Gossios et al. [1], water-enema multidetector CT (WE-MDCT) has developed into a technique dedicated to visualising the large bowel, which relies on a combination of preliminary bowel cleansing, retrograde fluid-attenuation luminal distension, pharmacological hypotonisation and intravenous contrast enhancement. Although relatively simple to perform and interpret, WE-MDCT is increasingly considered the most accurate imaging technique to comprehensively stage colorectal carcinoma (CRC) and has very high sensitivity (98.6–99%) for lesions measuring at least 1 cm and good agreement between CT features and histopathology [2–5]. Moreover, WE-MDCT has been effectively adopted to diagnose bowel endometriosis [6–8], colonic diverticular disease [9] and chronic inflammatory bowel diseases (IBD) [9–13].

Following colorectal surgery for either benign or malignant processes, optical colonoscopy remains the gold standard technique to assess the anastomotic site and residual large bowel and to identify recurrence of resected disease. However, in operated patients endoscopy is often hampered by postsurgical adhesions, sharp bowel angulations, bowel kinking, poor bowel preparation and anastomotic strictures (AS). WE-MDCT allows reliable measurement of the colonic wall thickness in normal and pathological conditions, and provides a detailed multiplanar assessment of mural, intra- and extraluminal abnormalities of the large bowel. Therefore, its use is appealing to investigate the operated colon, particularly after unsuccessful, incomplete or inconclusive endoscopy.

Based upon personal experience at a tertiary hospital that performs IBD and oncologic surgery, this pictorial essay presents the WE-MDCT technique and pitfalls, the expected appearances after various colon surgeries and the imaging features of benign anastomotic disorders, recurrent tumours and Crohn’s disease (CD).

## Water enema multidetector CT technique and interpretation

### Acquisition technique

Retrograde colonic distension is contraindicated in the presence of high-grade bowel obstruction, when a standard contrast-enhanced CT acquisition reliably investigates the site and cause of obstruction, thanks to the pre-existent bowel dilatation with intraluminal fluid. Before WE-MDCT, bowel cleansing is obtained using an iso-osmolar non-absorbable laxative solution (typically 4–6 doses of polyethylene glycol dissolved in 500 ml water per dose) the day before the examination, in association with a low-fibre diet for 3 days. Patients fast for 12 h after a liquid dinner the evening before the scheduled examination [9].

**Fig. 1:**
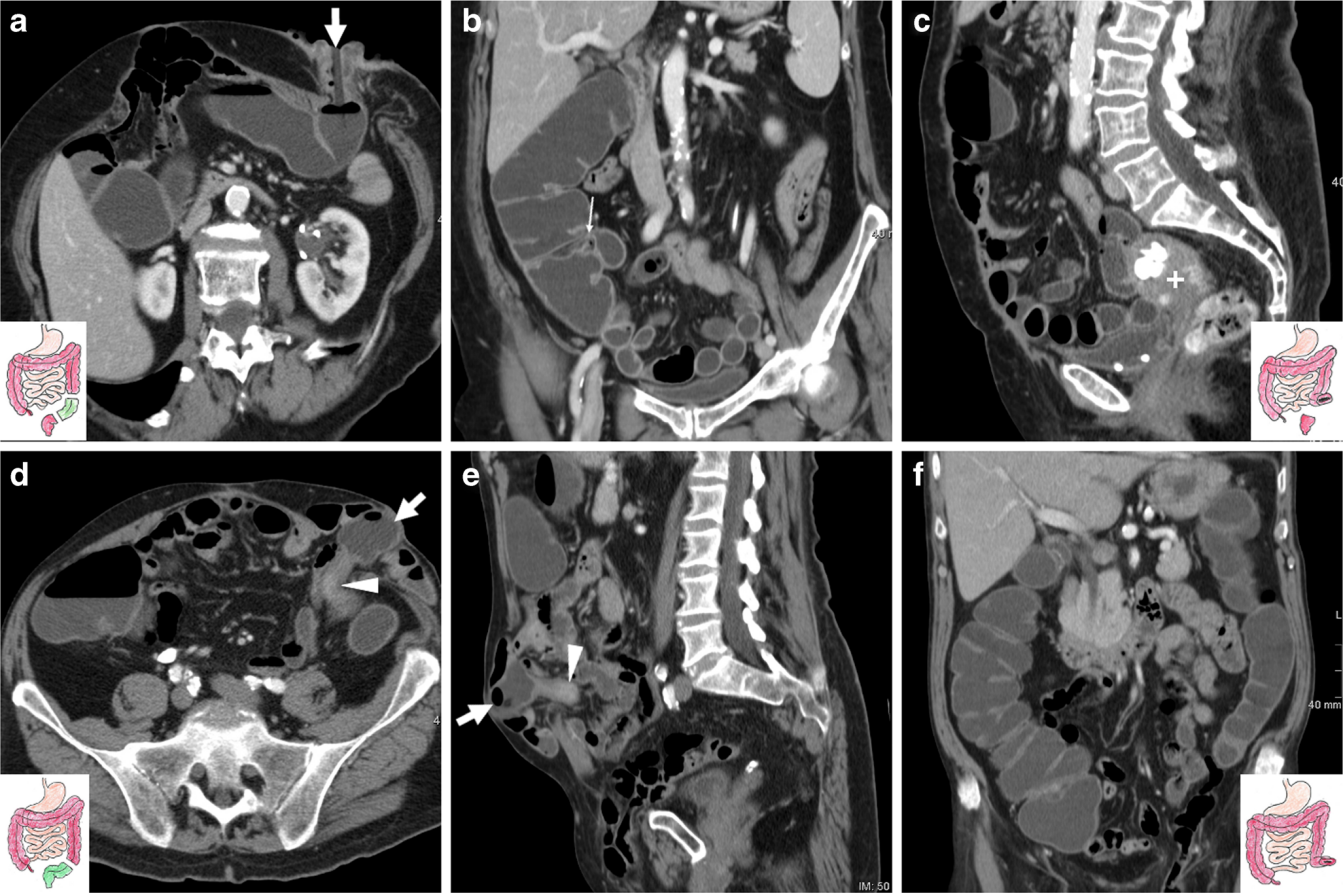
Two examples of water-enema multidetector CT (WE-MDCT) via the colostomy (surgical schemes shown in insets). **a**–**c** performed before elective bowel recanalisation in a 79-year-old female with previous Hartmann’s resection of the sigmoid and descending colon for complicated diverticulitis. Optimal retrograde distension of the residual large bowel up to the ileocecal valve (thin arrow in **b**) is achieved using a Foley catheter (thick arrow in **a**) positioned in the left-sided stoma. Note the closed rectal stump (+ in **c**). **d**–**e**) performed via the permanent colostomy in an 85-year-old male following abdomino-perineal resection for perforated rectal cancer (note absent rectum in **e**). Segmental poor distension of the pre-stomal tract (arrowheads in **d**, **e**) surrounded by stomal herniation of small bowel loops. Optimally distended descending, transverse and right colon (in **f**) without abnormalities

The patient is positioned on the CT scanner table and is instructed to turn to the left lateral decubitus. A lubricated enema tube is gently inserted into the rectum and connected to a bag that contains 1.5–2 l of warm tap water. Retrograde colonic distension through gravity is obtained within a few minutes and is stopped when the patient complains of abdominal distension. Compared with surgically naive patients, distension of the operated colon requires a reduced amount of water, depending on the extent of resection. Then, the patient is turned to his/her right side to improve water distribution in the colon and finally positioned supine for CT acquisition. During retrograde filling, pharmacological hypotonisation is induced with either 20 mg hyoscine buthylbromide i.v. or 1 mg glucagon i.m. to improve the patient’s comfort and obtain colonic wall distension. In patients with a colostomy WE-MDCT may be also performed using a Foley balloon catheter positioned and inflated into the stoma opening (Fig. 1). Volumetric CT acquisition of the abdomen and pelvis during a single breath-hold is performed during intravenous injection of 110–130 ml of non-ionic iodinated contrast medium using an automated power injection at a 2.5 ml/s flow rate, with a 75-s scan delay. The water enema is drained externally by placing the water bag on the floor before removing the patient from the CT scanner table. The radiation exposure from WE-MDCT is analogous to that of a standard contrast-enhanced CT study of the abdomen and pelvis of the same patient on the same scanner. The total examination time generally does not exceed 10–15 min [9].

### Rationale and comparison with other CT techniques

In patients with a history of partial colonic resection for CRC, Neri et al. used CT colonography (CTC) after incomplete endoscopy to assess the colonic mucosa and pericolic tissues searching for local recurrence, metachronous polyps and tumours. Although the residual colon was always entirely visualised with 100% sensitivity for AS, in their experience CTC findings were not sufficiently reliable to differentiate fibrotic from neoplastic strictures. Furthermore, in patients with right hemicolectomy retrograde insufflation of the anastomosed ileum may cause suboptimal distension of the residual colon [14].

Compared with full-dose contrast-enhanced CTC, WE-MDCT has a shorter learning curve for radiologists, is less cumbersome for the patient without the need for rotation from the prone to supine position and generates a lower radiation dose because it involves a single CT acquisition during contrast medium injection. Although no studies compared the comfort between the two different techniques, Ridereau-Zins et al. observed that the retrograde introduction of warm water was well, moderately and poorly tolerated by 86.2%, 12.2% and 1.7% of patients, respectively [2]. Furthermore, WE-MDCT does not seem to suffer from the occasional but potentially severe complications associated with air or carbon dioxide CTC, including a 0.04% perforation rate [15]. At our centre, the vast majority of patients do not experience side effects and consistently feel WE-MDCT is less disagreeable than those receiving colonic distension using litres of air [9]. After WE-MDCT, Paparo et al. reported four (1.3%) mild adverse events (nausea, abdominal discomfort) and one episode of diarrhoea in a cohort of 30 patients with a high proportion of underlying bowel disorders. In the same study, the frequency of side effects with WE-MDCT was the lowest compared with peroral CT enterography (CTE), CT enteroclysis with intubation and combined CTE plus WE-MDCT [16].

Although prospective trials comparing accuracy for lesion detection between CTC and WE-MDCT are lacking, some authors have compared the degree of colonic distension between the two techniques, concluding that the sigmoid and left colon were better assessed using both WE-MDCT and prone CTC compared with the supine CTC acquisition [13]. Therefore, we started to use WE-MDCT to assess diverticular disease and chronic inflammatory bowel diseases [9] and suspected abnormalities of surgical anastomoses. Together with the two leading French groups, we believe that—compared with CTC—retrograde filling using water does not overdistend the colonic lumen and thus allows a better assessment of the true mural thickness of the irregular external edges that define T3-stage CRC and of peritumoral lymph nodes [2–5].

In the setting of chronic IBD, WE-MDCT has been validated by the Genoa group, consistently provides superior distension of the large bowel compared with CTE and optimally reproduces the well-known mural and extraluminal features of CD [10–12]. Furthermore, without the patient having to ingest polyethylenglycole solution, WE-MDCT achieves adequate luminal filling of the neoterminal ileum in a high proportion of patients with ileocecal resection (ICR) [9–12].

### Interpretation

Similarly to the preoperative setting [17], interpretation of CT studies focused on the large bowel benefits from multiplanar image review: therefore, WE-MDCT images should be routinely reconstructed along the axial, coronal and sagittal planes. We suggest that radiologists should review the study on a workstation to save oblique or curved-planar reconstruction images focused on the key findings, such as surgical anastomoses and strictures.

Interpretation of WE-MDCT scans benefits from the excellent contrast between the intraluminal water, enhanced colonic wall and normal fat-attenuation perivisceral planes. In surgically treated patients, WE-MDCT allows depicting the anatomy and position of the residual large bowel in arbitrary planes, including the coronal orientation, which is most appealing for surgeons. Study interpretation benefits from precise knowledge of the type of resection and reconstruction performed and of recent endoscopic findings. Surgical anastomoses should be evaluated for site, configuration, patency and mural features. Ileo-colic and colo-colic anastomoses can be either manual (hand-sewn) or mechanical, with the latter clearly identified by the presence of hyperattenuating circular or linear staple lines. Focused maximum-intensity projection (MIP) images may be helpful to assess the configuration and integrity of a stapled anastomosis (Fig. 3, image c). Known or indeterminate strictures should be assessed for length, configuration, mural thickness, entity and pattern of enhancement (homogeneous or stratified) and associated extraluminal changes.

## Expected CT appearances after colorectal surgery

**Fig. 2:**
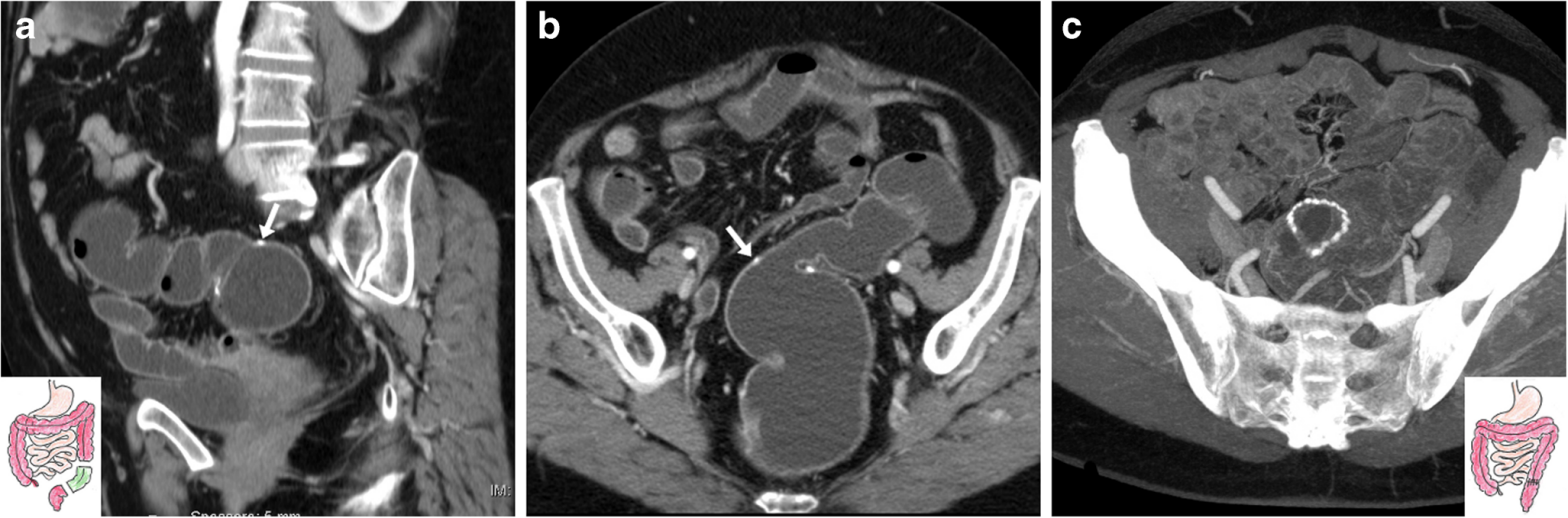
Expected status after elective sigmoid colon resection for diverticular disease with primary anastomosis (surgical scheme shown in insets) in a 60-year-old female. WE-MDCT (**a**, **b**) showed good distension of the rectum and upstream residual colon and patent colo-colic anastomosis (arrows). The stapled anastomosis (**c**) is depicted using maximum-intensity projection (MIP) reconstruction on the axial plane (**c**)

**Fig. 3:**
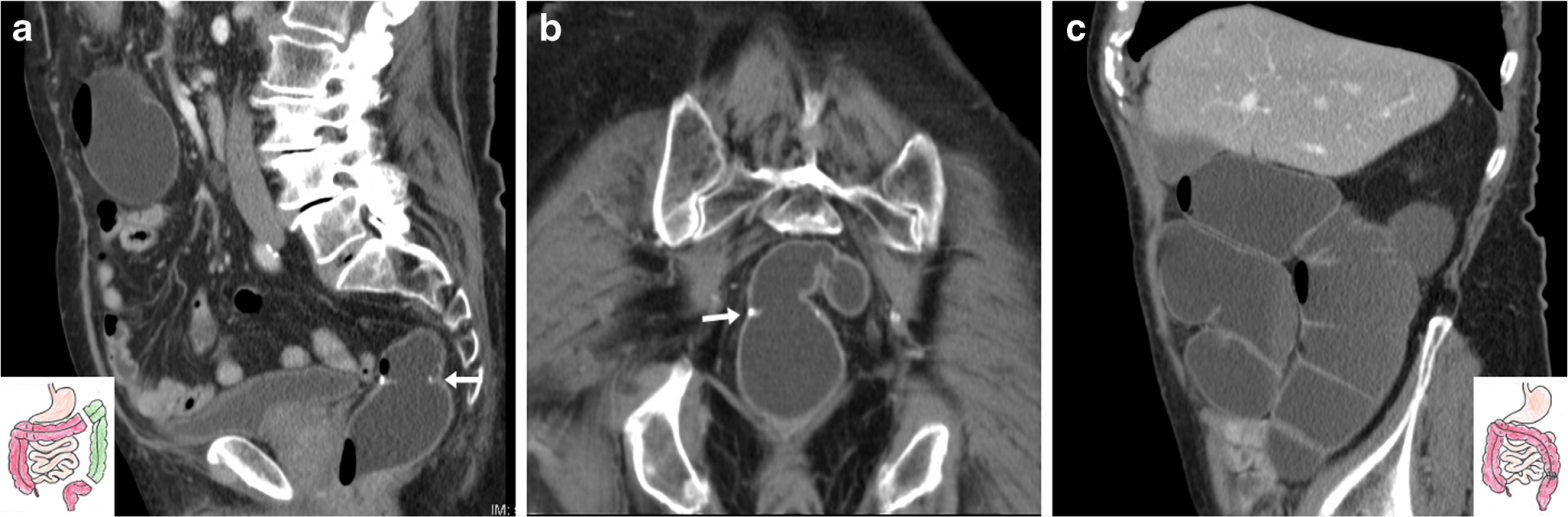
Expected status after left hemicolectomy (surgical scheme shown in insets) for carcinoma in an 86-year-old male with impassable anastomosis. Multiplanar WE-MDCT images (**a**–**c**) showed a normally distended rectum, patent stapled end-to-end colorectal anastomosis (arrows), well-distended hepatic flexure and right colon (in **c**) in the normal anatomic site

In the left-sided colon, colo-colic anastomoses are generally shaped end-to-end, since the bowel lumen has a similar diameter on both sides. In normal conditions, the colonic wall thickness at a surgical anastomosis should not exceed 3–4 mm (Figs. 2, 3). In a common clinical situation, WE-MDCT may be indicated before elective recanalisation 4–6 months following an urgent Hartmann’s procedure (HP) to treat complicated diverticulitis, obstructing or perforated CRC. In HP, segmental resection of the sigmoid or descending colon is completed with creation of a temporary diverting end colostomy to avoid the risk of anastomotic dehiscence, and a blind-rending rectal stump is left in place (Fig. 1). Following elective sigmoidectomy with primary reconstruction, WE-MDCT shows mild shortening of the large bowel and a patent end-to-end colo-colic anastomosis with normal mural thickness (Fig. 2). Anterior rectal resection (ARR) involves removal of the distal sigmoid colon, recto-sigmoid junction and most of the rectum and may be reconstructed with either a coloanal (in low ARR) or colorectal anastomosis with the preserved terminal rectum. After standard (Fig. 3) or extended left colectomy, a long colonic segment is removed, extending from the sigmoid to the splenic flexure or transverse colon respectively, with the hepatic flexure at its normal site [18–20].

**Fig. 4:**
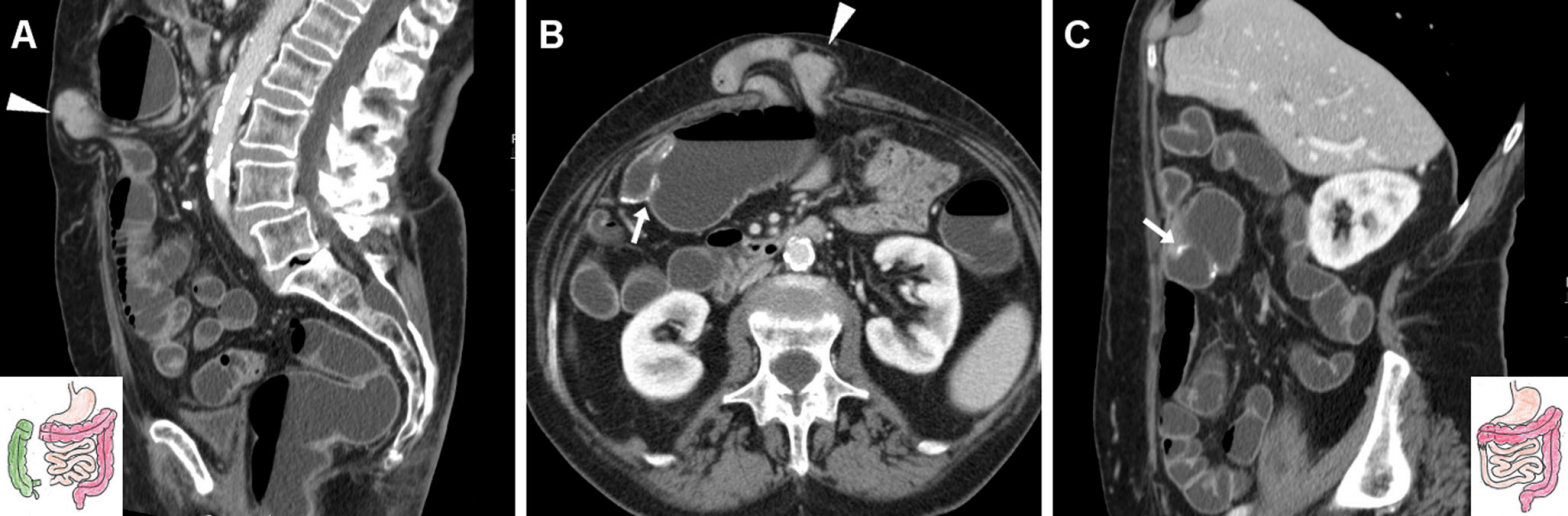
Expected status after right hemicolectomy (surgical scheme shown in insets) for carcinoma in an 88-year-old female. WE-MDCT showed well-distended residual bowel and patent end-to-side ileo-colic anastomosis (arrows) with hyperattenuating staples. Note the midline incisional hernia containing small bowel (arrowheads)

**Fig. 5:**
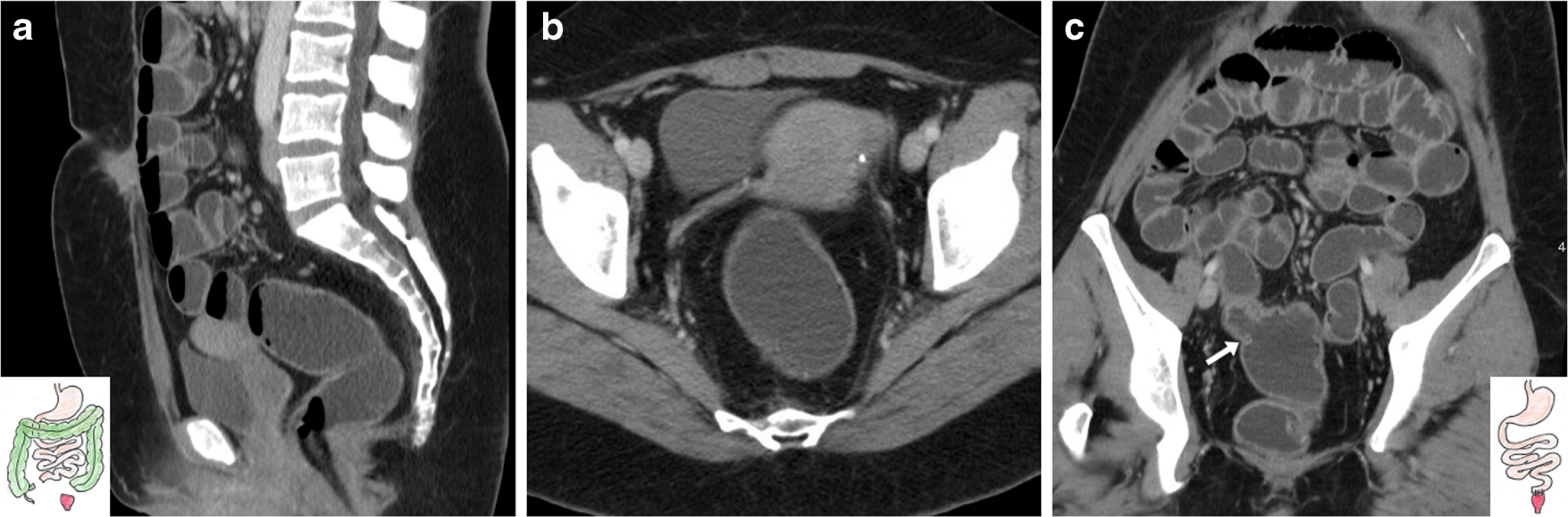
Expected postoperative status following subtotal colectomy (surgical scheme shown in insets) in a 45-year-old female with ulcerative colitis. WE-MDCT (**a**–**c**) showed a well-distended rectum with thin walls, normal mesorectal space, patent end-to-side ileo-rectal anastomosis (arrow in **c**) and distended jejuno-ileal loops

End-to-side anastomoses are used when a smaller proximal lumen is connected to a larger one such as after standard (Fig. 4) or extended right colectomy: in these cases WE-MDCT shows absence of the right colon from the terminal ileum through the hepatic flexure or transverse colon, respectively. Moreover, subtotal colectomy (indicated to treat ulcerative colitis, fulminant infectious colitis, synchronous malignancies and hereditary polyposis syndromes) may be completed with either a permanent or temporary ileostomy or an end-to-side anastomosis between the preserved rectum and distal ileum (Fig. 5) [18–20].

**Fig. 6:**
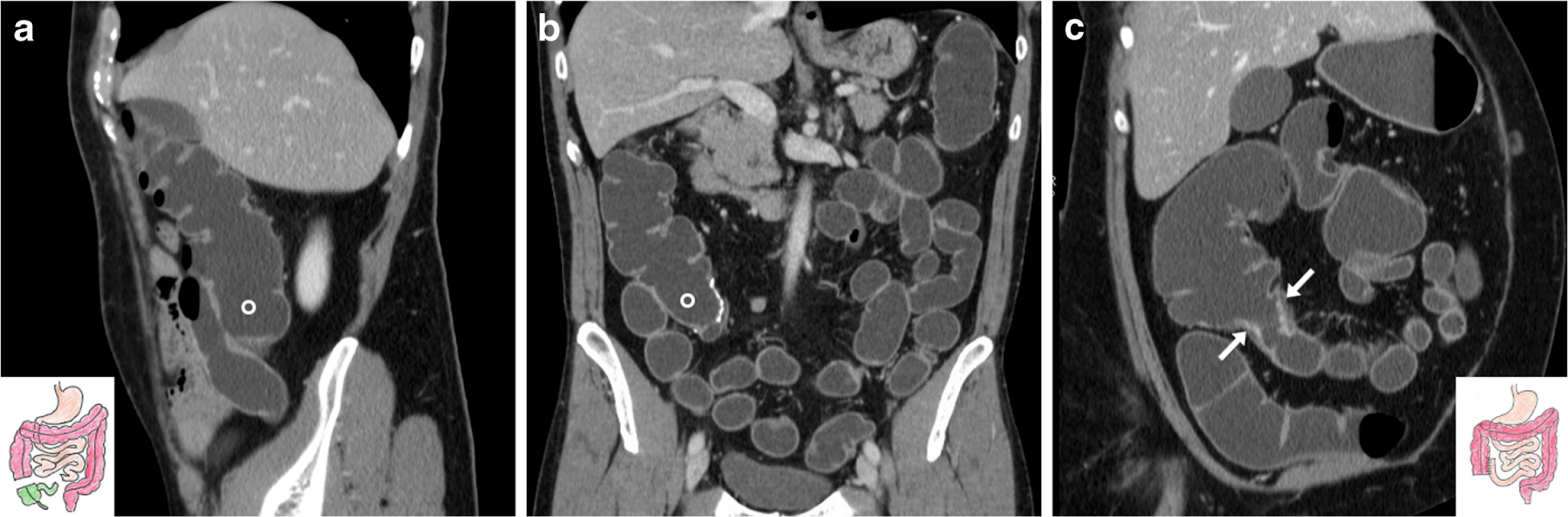
Expected status after ileocecal resection (surgical scheme shown in insets) for Crohn’s disease (CD) complicated by mesenterial abscess. In keeping with normal endoscopic findings, WE-MDCT (**a**–**b**) showed good distension of the proximal residual colon up to the distal cul-de-sac (o) and of the neoterminal ileum. Focused oblique reconstruction (**c**) showed patent side-to-side ileo-colic anastomosis (arrow) with non-thickened walls and minimal mucosal hyperaemia

**Fig. 7:**
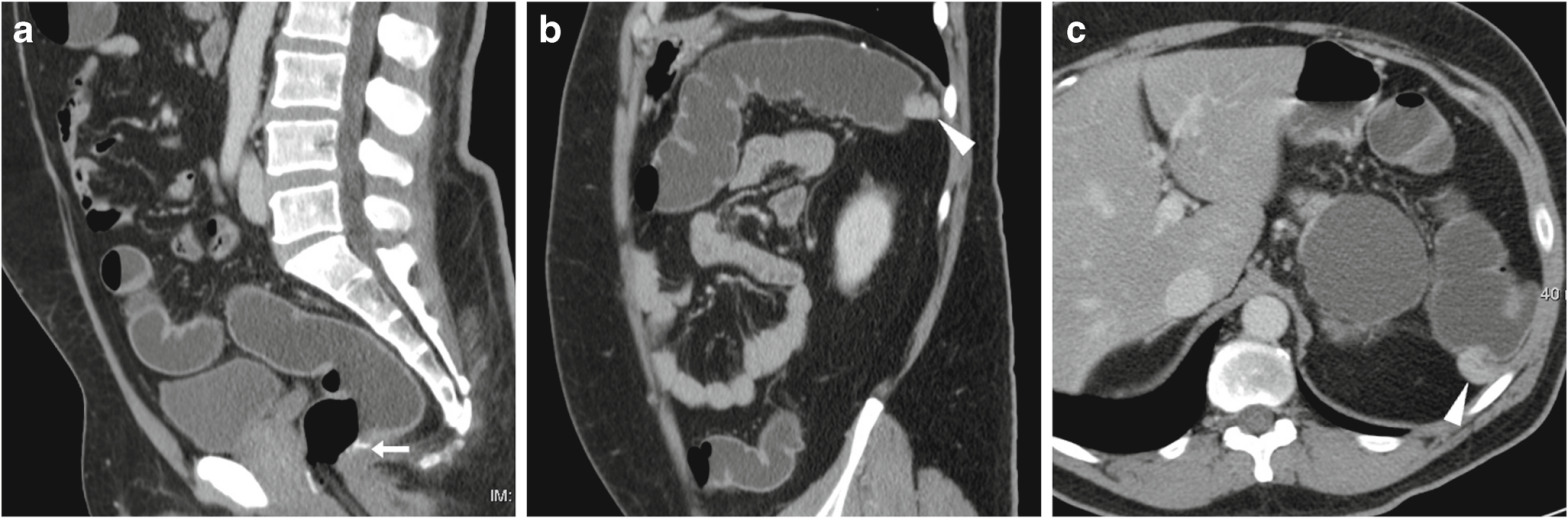
Assessment of postsurgical positioning of large bowel in a 40-year-old male with rectal prolapse repair, history of splenectomy and distal pancreatectomy for neuroendocrine tumour. Following incomplete colonoscopy, WE-MDCT showed a normally positioned and distended rectum with suture (arrows) and very thin presacral space; left colon flexure occupying the site of splenectomy, indissociable from splenosis nodules (arrowheads) unchanged from previous studies

Following ICR for CD, the ileocolic anastomosis is frequently of the end-to-side or side-to-side type with a distended, prominent colonic blind end (“cul-de-sac”) (Fig. 6). Finally, WE-MDCT is also useful to assess the large bowel after extra-colonic abdominal and pelvic surgeries that may cause repositioning, extrinsic compression of the colon or both (Fig. 7) [18–20].

## Local anastomotic complications

The development of a benign colorectal AS is an increasingly common postsurgical problem. Strictures may form after open or laparoscopic surgical treatment of either benign or malignant diseases, with a higher incidence in stapled compared with hand-sewn anastomoses. Although causes are still not clearly understood, AS may result from ischaemia, disruption or leakage at the anastomotic site. Symptoms such as pain, distension, nausea and vomiting are reported in up to one-third of patients after colorectal surgery and reflect the degree of luminal stenosis [21–23].

**Fig. 8:**
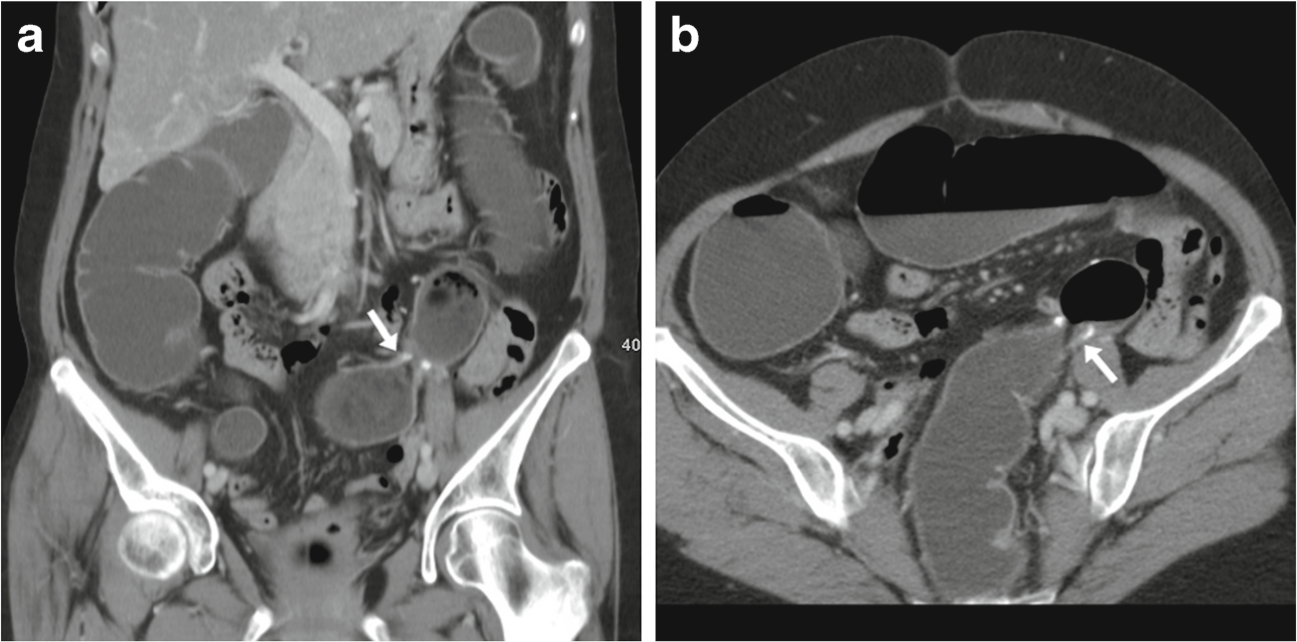
Fibrotic anastomotic stricture in a 63-year-old female with previous sigmoid colon resection and endoscopically impassable anastomosis. WE-MDCT showed hourglass-shaped colo-colic anastomosis (arrows) with normal mural thickness, without adjacent abnormalities, and well-distended upstream large bowel

**Fig. 9:**
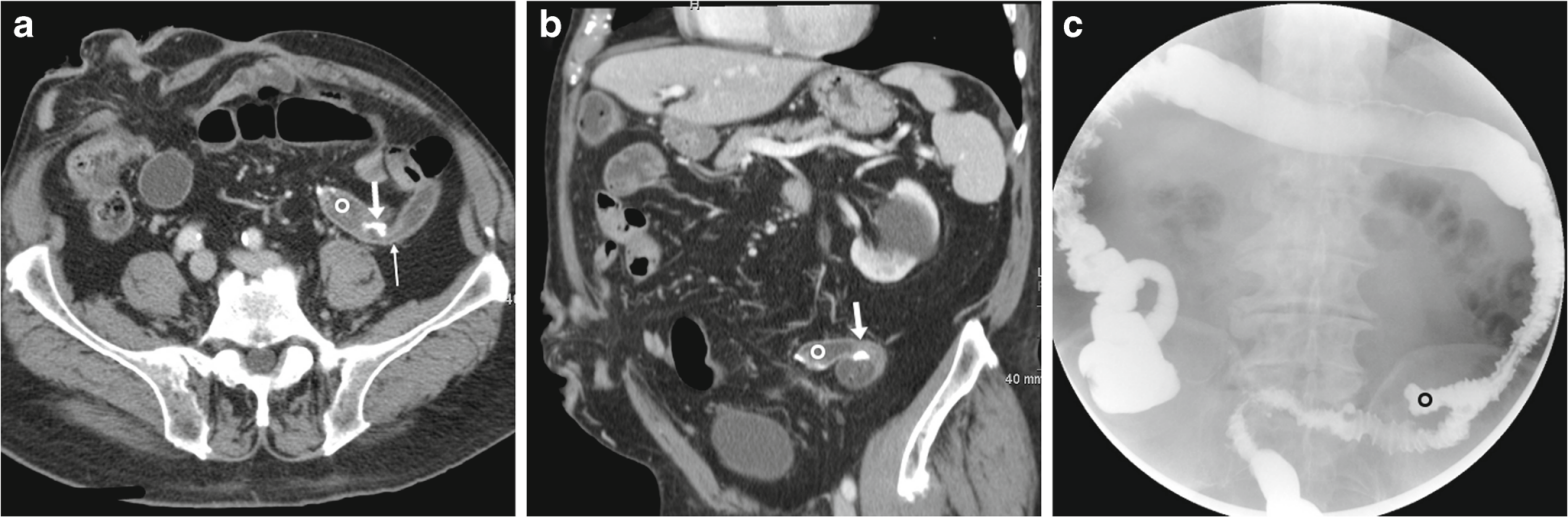
Anastomotic recess and kinking in an 87-year-old male with a history of left hemicolectomy for descending colon carcinoma, complicated by anastomotic dehiscence. WE-MDCT (**a**–**b**) showed colo-colic anastomosis with staples (arrows), a blind-ending recess (o) and abrupt kinking (thin arrows). The recess (o) persisted on Gastrografin enema (**c**) performed to exclude iatrogenic perforation following pneumatic anastomotic dilatation

The characteristic appearance of a benign, fibrotic AS includes an indistensible, often hourglass-shaped colo-colic anastomosis with normal mural thickness, without adjacent abnormalities (Fig. 8). Alternatively, an angulated anastomosis (Fig. 9) may underlie similar manifestations [24].

In our experience, WE-MDCT is particularly helpful when repeated surgery or endoscopic treatment of AS is being contemplated. Balloon dilatation with or without endoscopic incision using laser or argon plasma is successful in 88% of benign AS cases. Alternatively, refractory ASs are increasingly treated with fully covered stents to achieve a prolonged clinical success and to obviate the high morbidity from revisional surgery [22, 23, 25–28].

**Fig. 10:**
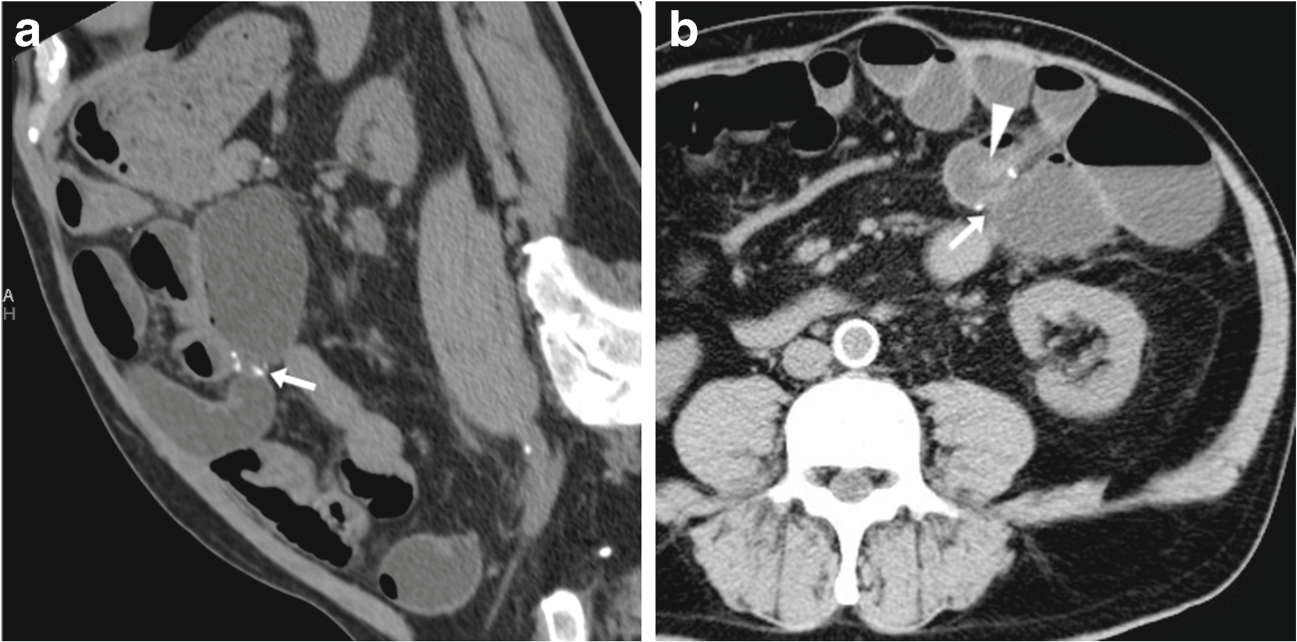
Anastomotic ulcer in a 78-year-old male with history of extended right hemicolectomy for pT3N1 colon carcinoma. Previous endoscopy revealed irregularly hyperaemic side-to-side ileo-colic anastomosis with biopsy findings indicating severe acute and chronic inflammation. Performed without intravenous contrast because of impaired renal function, WE-MDCT showed moderately tight stapled anastomosis (arrows), with focal intraluminal vegetation (arrowhead) limited to the ileal side. Repeated endoscopy diagnosed anastomotic ulcer, stable at 6-month follow-up

Inflammatory anastomotic ulcers of unknown cause may occasionally develop at ileo-colic anastomoses months to years after the primary surgery. These rare conditions generally manifest with occult blood loss, do not respond to anti-inflammatory, antibiotic and immunosuppressive medications, and frequently recur after surgical treatment. At WE-MDCT, focal irregularities or unspecific intraluminal projections (Fig. 10) may be seen [29, 30].

## Neoplastic recurrences

Following CRC resection, patients are at risk for local anastomotic or extraluminal tumour recurrence and may develop adenomatous polyps and metachronous tumours in the residual bowel. At endoscopic surveillance, abnormal findings are found within 2 years from surgery in up to 18.5% of patients [31, 32]. Recurrent malignancies generally require repeated surgery or palliative endoscopic stenting [24, 25].

**Fig. 11:**
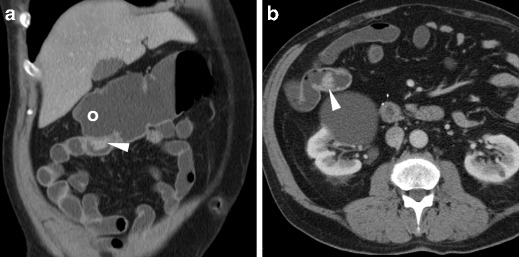
Intraluminal neoplastic recurrence at the anastomotic site in a 66-year-old male with previous right hemicolectomy. Follow-up WE-MDCT showed side-to-side ileo-colic anastomosis with a blind-ending colonic recess (o) and a 2-cm solid, enhancing vegetation (arrowheads): endoscopy and biopsy confirmed recurrent carcinoma

**Fig. 12:**
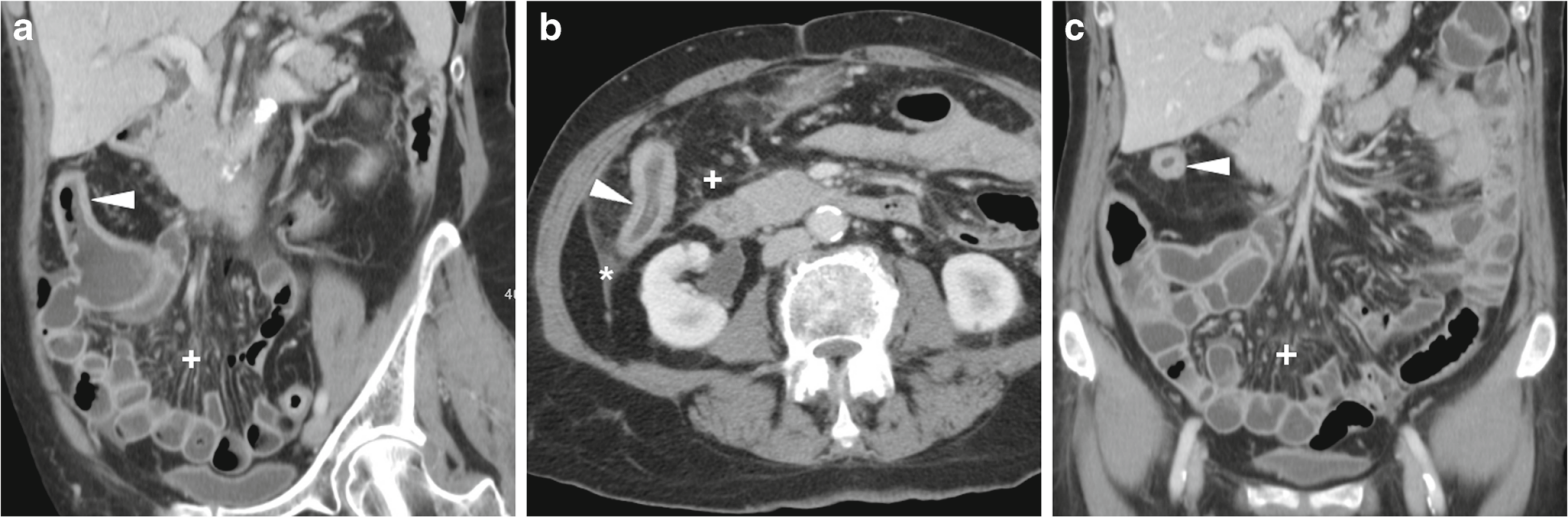
Serosal and intramural neoplastic recurrence in a 74-year-old female with a history of left hemicolectomy for G3 signet-ring colon carcinoma. Corresponding to an endoscopically impassable stricture, WE-MDCT (**a**–**c**) showed a poorly distended segment with thickened walls and non-stratified enhancement (arrowheads) located just distal to the ileo-colic anastomosis, minimal peritoneal effusion (* in **b**) and mesenteric fat stranding (+) with centimetric lymphadenopathies. Final diagnosis was intra-abdominal carcinomatosis

**Fig. 13:**
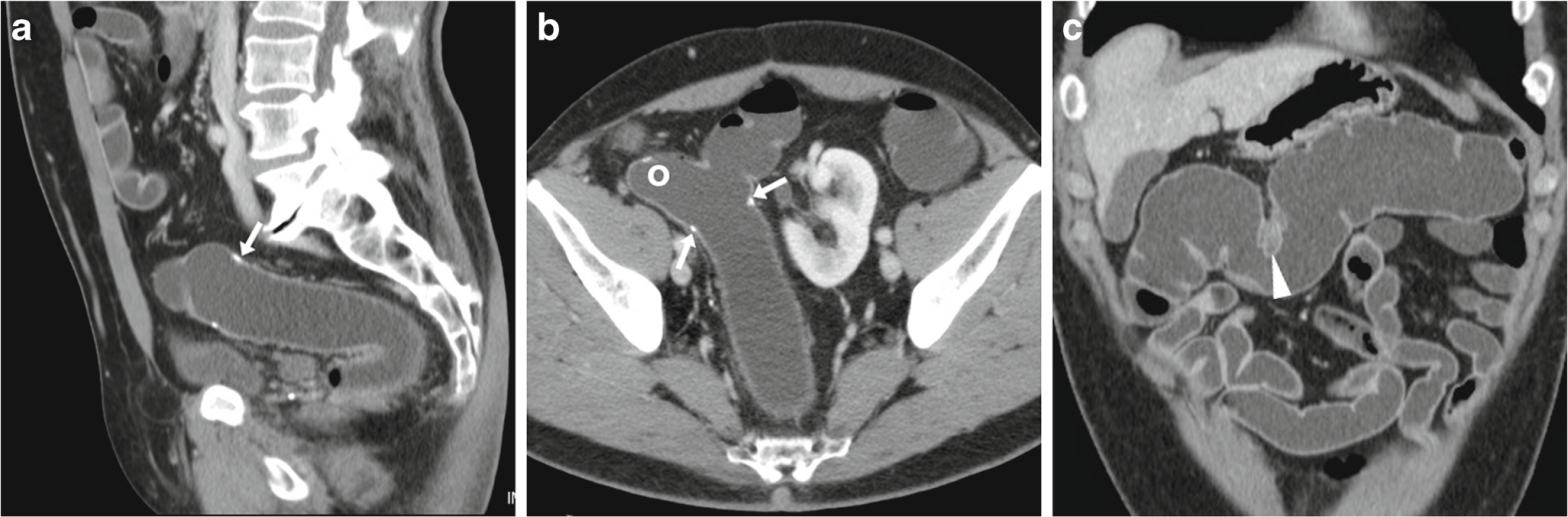
Metachronous polyp in the residual colon 10 years after left hemicolectomy for carcinoma. WE-MDCT (**a**–**c**) showed normal colo-colic anastomosis (arrows) with a blind-ending recess (o) and a 1.5 cm polyp (arrowhead in **c**) attached to a haustral fold in the transverse colon, which required endoscopic resection

The hallmark of a local neoplastic recurrence is a solid, more or less homogeneously enhancing mass that projects in the lumen (Fig. 11) or abuts the external (serosal) aspect of the colonic anastomosis. Alternatively, recurrent tumour may appear as a stricturing segment with marked, sometimes asymmetric wall thickening with solid attenuation and positive contrast uptake (Fig. 12). Furthermore, in patients with a history of resected CRC WE-MDCT may detect metachronous polyps (Fig. 13) or tumours in the residual bowel.

## Crohn’s disease recurrences

**Fig. 14:**
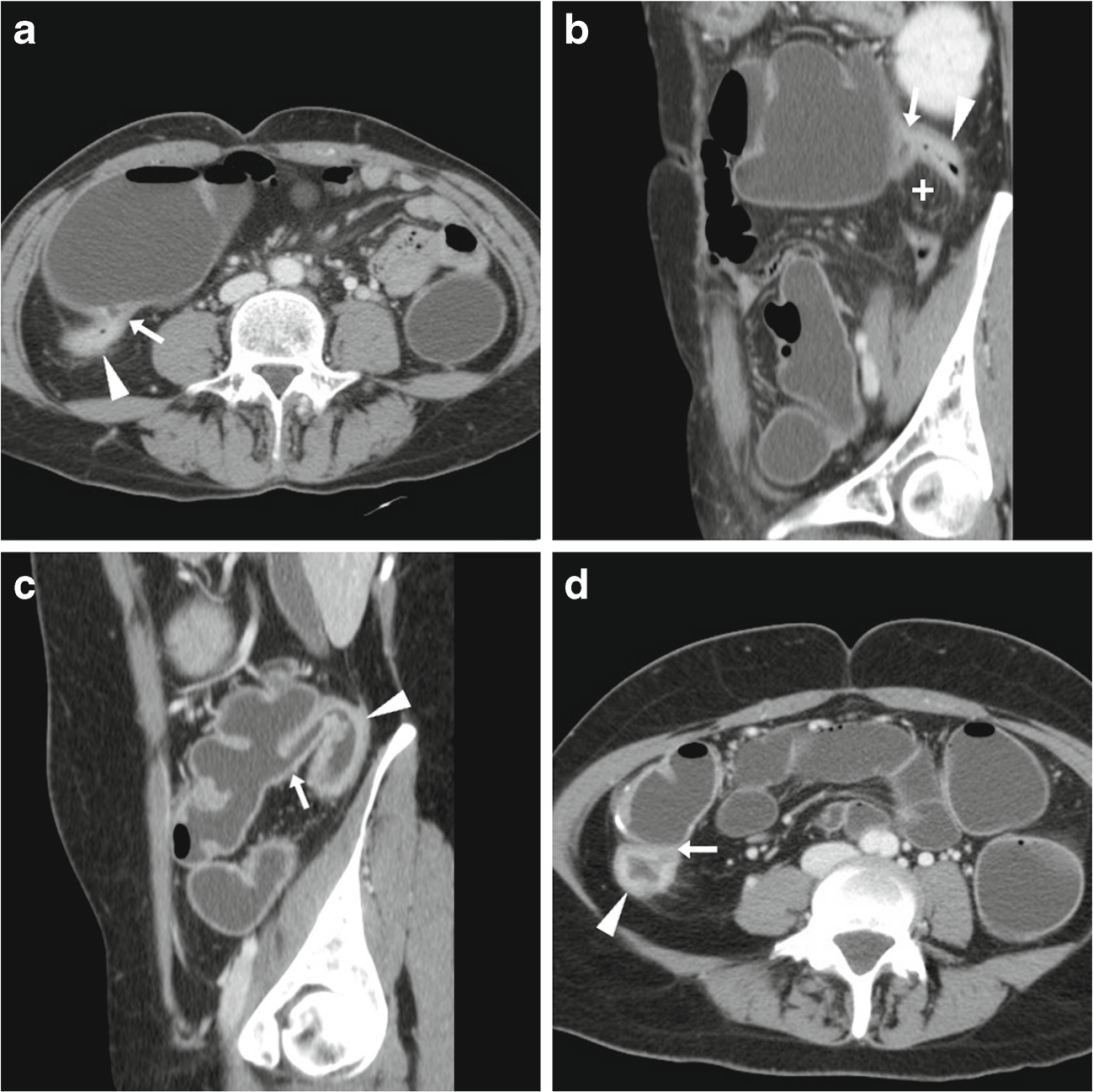
Two cases of recurrent CD after ileo-colic resection. **a**–**b** Proximally to the ileo-colic anastomosis (arrows), the neoterminal ileum (arrowheads) does not distend retrogradely during WE-MDCT and shows thickened walls with enhancing mucosa and perivisceral vascular engorgement (“comb sign”, +). **c**–**d** Proximally to the well-distended colon, the ileo-colic anastomosis (arrows) appears moderately patent (arrows). Recurrent CD is depicted as segmental, homogeneously enhancing mural thickening (arrowheads) with mucosal irregularities

**Fig. 15:**
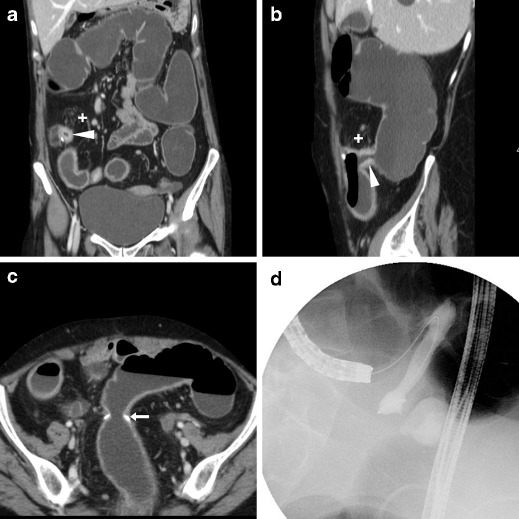
Differentiation between CD recurrence at the ileo-colic anastomosis (arrowheads in **a**–**b** with similar appearance to Fig. 15) and fibrotic stricture at the site of previous sigmoid colon \resection (arrow in **c**, note similarity with Fig. 9): the latter was treated with balloon dilatation under fluoroscopy (**d**)

Following ICR, CD recurrence at the ileo-colic anastomosis occurs in up to 70% of patients. In this situation, the use of CT has a complementary role to endoscopy to assess the transmural and longitudinal extent of transmural inflammation and detect possible extramural complications of penetrating CD. Like with peroral CT enterography, the hallmark of relapsing CD is stratified mural thickening at the neoterminal ileum with either mucosal or homogeneous transmural hyperenhancement compared with other adjacent bowel loops, with associated prominence of mesenteric vessels (“comb sign”) (Figs. 14, 15A–B). Although the same findings may be depicted by MR enterography without using radiation, in operated patients the assessment of a stapled anastomosis may be limited by metallic artefacts. Furthermore, WE-MDCT easily depicts segmental involvement of the residual colon alternating with spared “skip” regions and of extraluminal features of CD. Therefore, in selected CD patients WE-MDCT may be helpful in the differentiation of fibrotic AS amenable to endoscopic treatment from active CD, which requires medical therapy (Fig. 15), and from chronic fibrostenosing CD with poor, homogeneous and non-stratified mural enhancement [9, 12].

## Technical pitfalls

**Fig. 16:**
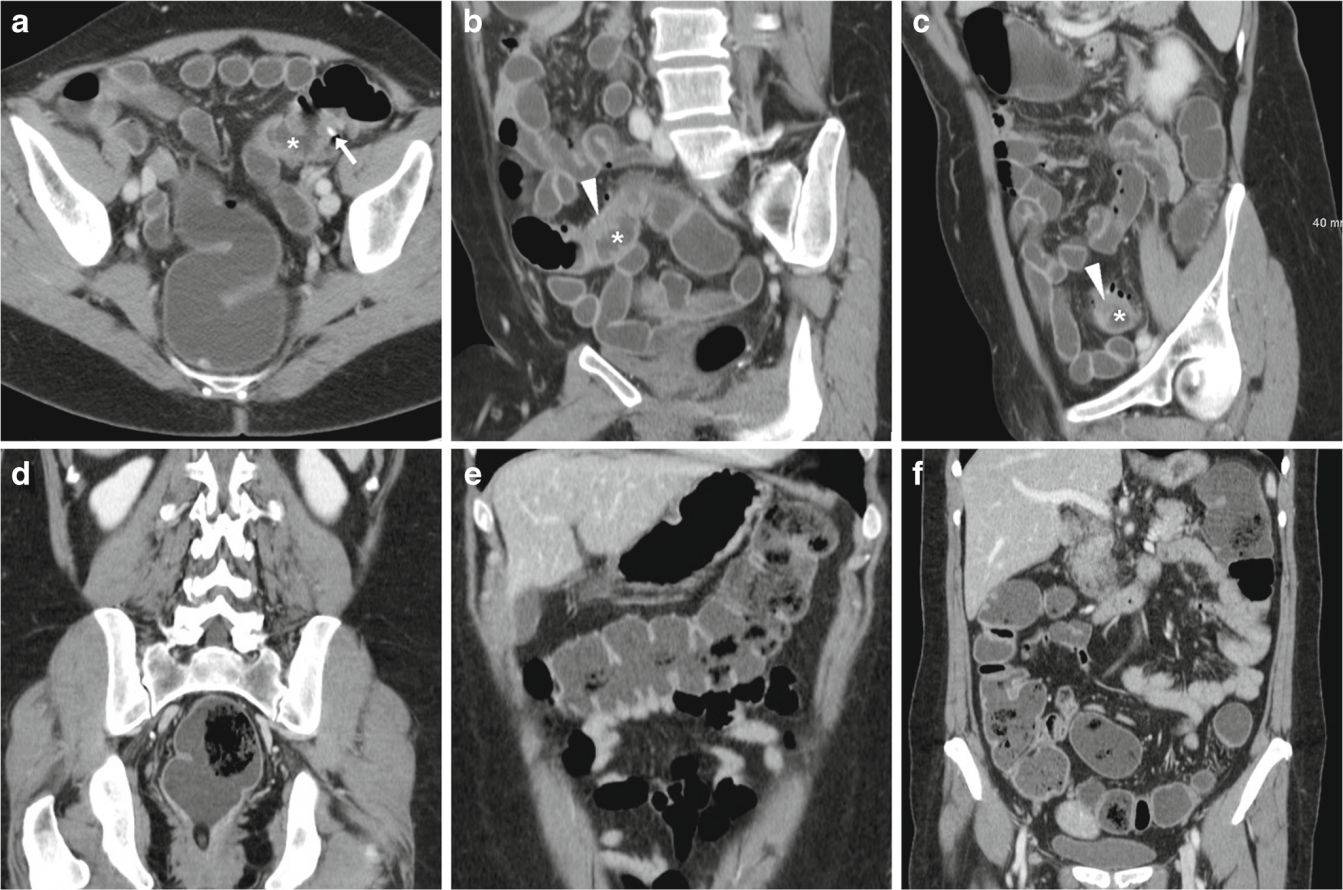
Common pitfalls of WE-MDCT. **a**–**c** Localised poor distension of the proximal sigmoid colon (*) with apparent eccentric mural thickening (arrowheads) in a 61-year-old female with history of iatrogenic perforation during polypectomy treated with endoscopic clipping (arrow in **a**). Clinical and endoscopic follow-up was unremarkable. **d**–**e** Poor bowel cleansing with sparse intraluminal faecal residues from the rectum (in **d**) throughout the well-distended colon in a 68-year-old female with history of polypectomy. Subsequent endoscopic follow-up was also hampered by incomplete cleansing

The three main pitfalls of this technique include:

1. failure to achieve retrograde colonic distension because of incontinence at either the rectum or colostomy;
2. segmental non-distension or spasm, which most commonly occurs at the sigmoid or the nondependent transverse colon and generally mimics a segmental mural thickening (Fig.16A–C); this phenomenon often disappears with further water inflow and repeated hypotonisation
3. presence of faecal residues from insufficient bowel cleansing (Fig.16D–E), which may obscure intraluminal processes but generally does not impede identification of strictures and assessment of mural thickness.

## Conclusion

In our experience, WE-MDCT is a rapid, easy-to-perform and well-tolerated technique that provides multiplanar high-resolution visualisation of the operated large bowel, with the drawback of ionising radiation. In selected patients with a history of colorectal surgery, WE-MDCT may prove useful as a complementary technique to endoscopy to assess suspected abnormalities at the anastomosis. Furthermore, WE-MDCT allows assessing the calibre, length, mural and extraluminal features of luminal strictures and evaluating the bowel upstream to an endoscopically impassable tract and is therefore particularly helpful when endoscopic procedures or surgical interventions are being planned.
